# End to End Digitisation and Analysis of Three-Dimensional Coral Models, from Communities to Corallites

**DOI:** 10.1371/journal.pone.0149641

**Published:** 2016-02-22

**Authors:** Luis Gutierrez-Heredia, Francesca Benzoni, Emma Murphy, Emmanuel G. Reynaud

**Affiliations:** 1 School of Biology & Environmental Science, UCD Science Centre, University College Dublin, Belfield, Dublin, Ireland; 2 Department of Biotechnology and Biosciences, Università degli Studi di Milano-Bicocca, Milano, Italy; Università di Genova, ITALY

## Abstract

Coral reefs hosts nearly 25% of all marine species and provide food sources for half a billion people worldwide while only a very small percentage have been surveyed. Advances in technology and processing along with affordable underwater cameras and Internet availability gives us the possibility to provide tools and softwares to survey entire coral reefs. Holistic ecological analyses of corals require not only the community view (10s to 100s of meters), but also the single colony analysis as well as corallite identification. As corals are three-dimensional, classical approaches to determine percent cover and structural complexity across spatial scales are inefficient, time-consuming and limited to experts. Here we propose an end-to-end approach to estimate these parameters using low-cost equipment (GoPro, Canon) and freeware (123D Catch, Meshmixer and Netfabb), allowing every community to participate in surveys and monitoring of their coral ecosystem. We demonstrate our approach on 9 species of underwater colonies in ranging size and morphology. 3D models of underwater colonies, fresh samples and bleached skeletons with high quality texture mapping and detailed topographic morphology were produced, and Surface Area and Volume measurements (parameters widely used for ecological and coral health studies) were calculated and analysed. Moreover, we integrated collected sample models with micro-photogrammetry models of individual corallites to aid identification and colony and polyp scale analysis.

## Introduction

It has been estimated that coral reefs support an approximate 25% of all marine life, and as a consequence, they are a significant food source for half a billion people worldwide [1]. The total economic value of these valuable organisms ranges from US$ 100,000 to US$ 600,000 per square kilometre per year [2]. They are crucial in supporting human life, but they are fragile and currently under serious hazard [3]. The decline in coral reef populations has been observed in recent decades, and it has been noted that up to 70% of the world's reefs are endangered or destroyed, partly by environmental stress but mostly due to human activity [4, 5].

Globally, coral reefs occupy an estimated area of 284,000 km^2^ [6]. However, monitoring efforts and biodiversity surveys have been relatively scarce and sparse, based largely in hot spots, reefs with nearby established scientific stations and areas constantly distressed by tourist activities. Many known reefs have not been surveyed and some are considered 90% unmapped [7]. Moreover, classical monitoring efforts of coral reef benthic assemblages (quadrats, transects, manta tows, etc.) can be a very labour intensive task. They can rely on hundreds of volunteer divers, are error-prone, focused on a small/medium scale, can be dangerous to divers and have a considerable carbon foot print [8]. If accurate and precise methods are used, monitoring becomes very time consuming [9]. Coral research can also be destructive, as some studies require a high number of colonies or segments of them [one study sampled up to 72 colonies) for treatment and analysis [10]. Furthermore, if non-contact measurement methods are employed, visual estimates performed by SCUBA divers are not accurate enough to detect small dimensional changes and inter-observer variability increases errors [11]. Reef-scale remote sensing technologies have been effectively used in the last decades, like satellite and LiDAR imaging [12, 13, 14, 15], but these have inconsistencies in accuracy (particularly in finer scale morphologies of coral colonies) [16]. These also have disparities in discrimination of distinct coral reef habitat boundaries, especially when the water column characteristics are not optimal, and can be costly and not readily accessible [17, 18].

Presently, the explosive development of electronic and imaging systems, and increasing internet speeds and computational power at a constantly reducing cost, have led to the inclusion of these technologies in survey and monitoring efforts. Several manufacturers have recently produced and released new lightweight devices which are easy to handle (e.g. GoPro), capable of performing under extreme conditions, providing low cost still images and video sequences of high resolution. These devices, coupled with intense development in visual and algorithm processing sciences along with companies competing to offer open source programs, have produced a viable alternative to traditional methods. They are easy to use without extensive design/programming nor computational skills, and are effective for morphological measurements.

Recently, a growing number of studies have investigated the use of 3D technologies. These involve photogrammetric approaches for underwater measurements of organisms and reef benthic assemblages, considering length, surface area, and volume calculations using still images or video [8, 19, 20, 21, 22, 23, 24]. However, those who have used stereo-photogrammetry usually have needed a bulky frame attached to the cameras to image underwater. This contraption can be mounted on a Remotely Operated Vehicle (ROV) or an Autonomous Underwater Vehicle (AUV) [25]. These techniques have also needed considerable programming and technical skills for calibration and processing. Some studies have relied on multi-image photogrammetry based monocular cameras to produce 3D models for their calculations [9, 26, 27, 28, 29, 30, 31, 32], although almost none of these studies produced 3D models with fine definition (intracolony or polyp scale) and quality texture mapping at this scale, nor sets of 3D constructs across different scales. A study proposed the novel capacity of *in situ* digitisation of 3D models based on Visual Odometry [9]. To the knowledge of the authors in this paper, there is only study done on corals using this specific software based on Structure From Motion (123D Catch, AutoDesk) [33]. This study performed dry and underwater estimations of Surface Area (SA) and Volume (V) on 3D models based on 3 growth forms of corals. The coefficient of variance and correlations of accuracy between sizes, shapes and object posture was also calculated using 3D models digitised using stills and videos from 3 different off-the-shelf cameras.

We propose a practical end-to-end, low-cost, non-intrusive, repeatable *in situ* method for producing multi-scale sets of 3D models of underwater coral colonies with corresponding 3D models of dry coral samples (needed for accuracy analysis). Physical parameters (e.g. surface area, volume, rugosity, etc.) were be estimated from these and high quality texture mapping can complement these calculations for other studies (corallite morphology, identification, living tissue, polyp density, epiphyte colonization, etc.). These 3D models are produced using off-the-shelf equipment and freeware user-friendly software needing low technical skills. 3D models generated from this technique can be used for analysis in biological surveys, monitoring, taxonomical research, online exchange for accessibility, virtual simulations, included in digital displays for education purposes and in 3D printing.

## Materials and Methods

The Ministry of Fisheries and Agriculture of the Republic of Maldives is the authority that issued the permits for sampling and the approval of the field trip. This was done in collaboration with the University of Milano-Bicocca Marine Research and High Education Centre in Magoodhoo.

### Coral sampling

**Fig 1:**
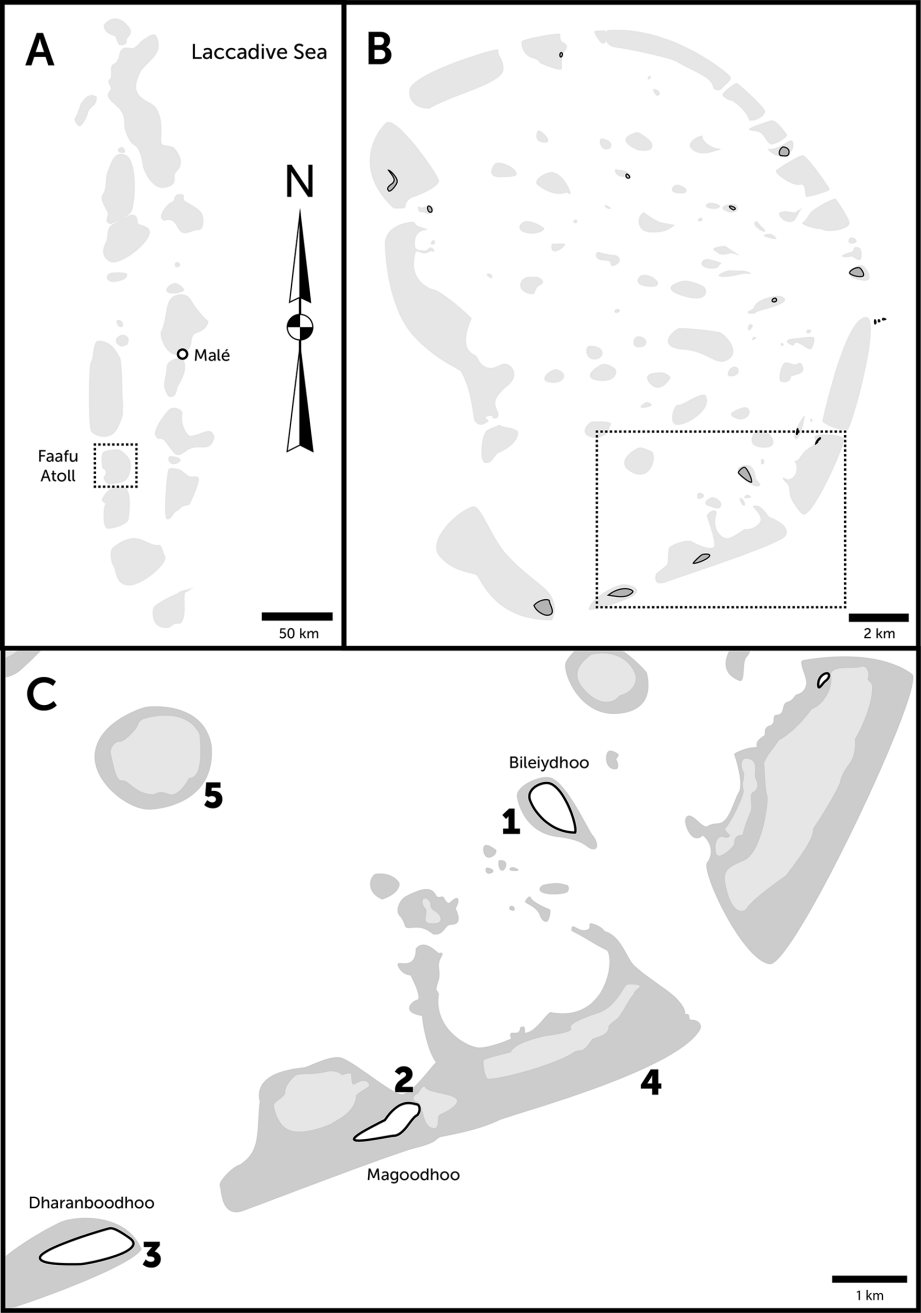
Geolocation of diving sites. A) Geolocation of Faafu Atoll in the Maldives. B) Geolocation of Magoodhoo island in the Faafu Atoll. C) Diving sites and coral reefs in the vicinity of Magoodho island. Sampled sites: 1. Wall Street 2. Magoodhoo Home Reef, left side 3. Dharanboodhoo Reef Bis 4. Dhigu Reef 5. Maaga.

Corals were imaged and sampled from 5 diving locations around Magoodhoo island, located in the Faafu Atoll, in Maldives (Fig 1A and 1B) in May 2014 using SCUBA diving equipment. The diving sites were Wall Street (N 03°07'14.2" E 72°58'46.9"), Magoodho Home Reef (N 03°03'40.6" E 72°57'57.5"), Daranboodho Reef Bis (N 03°03'40.6" E 72°55'52.1"), Dhigu Reef (N 03°04'48.9" E 72°58'57.7") and Maaga (N 03°07'11.5" E 72°56'21.7”) (Fig 1C).

### Examined corals

**Fig 2:**
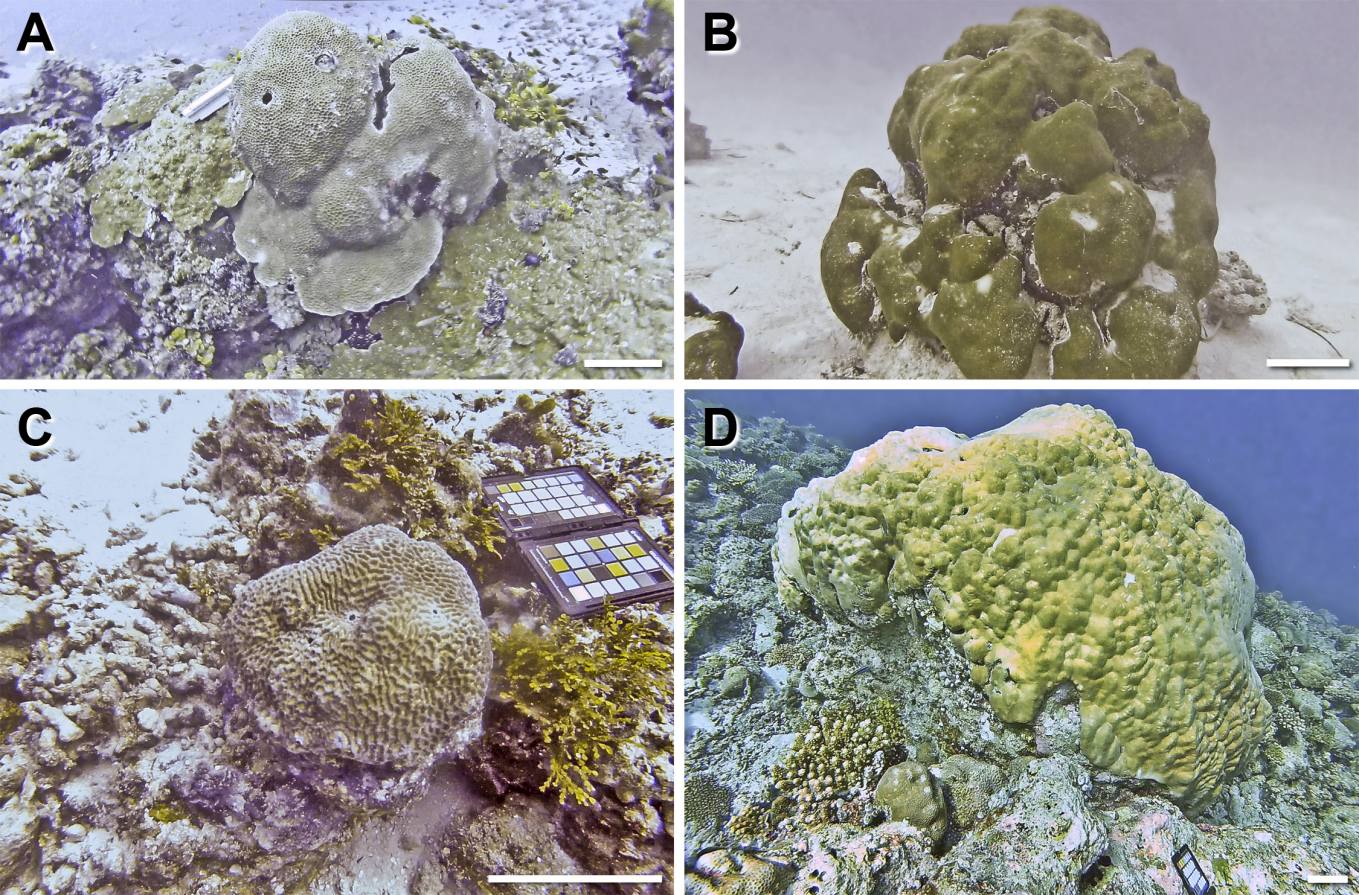
Massive coral growth forms sampled in this study (specie, location, date sampled). A) *Gardineroseris planulata*, Wall Street, 08/05/14 B) *Porites lutea*, Home Reef, 08/05/14 C) 8. *Platygyra daedalea*, Dhigu, 13/05/14 D) *Porites lobata*, Darambodhoo, 12/05/14. Scale bar 10 cm. Note: The coloured chart in some of the images is the ColorChecker Passport used for colour calibration and scale reference.

**Fig 3:**
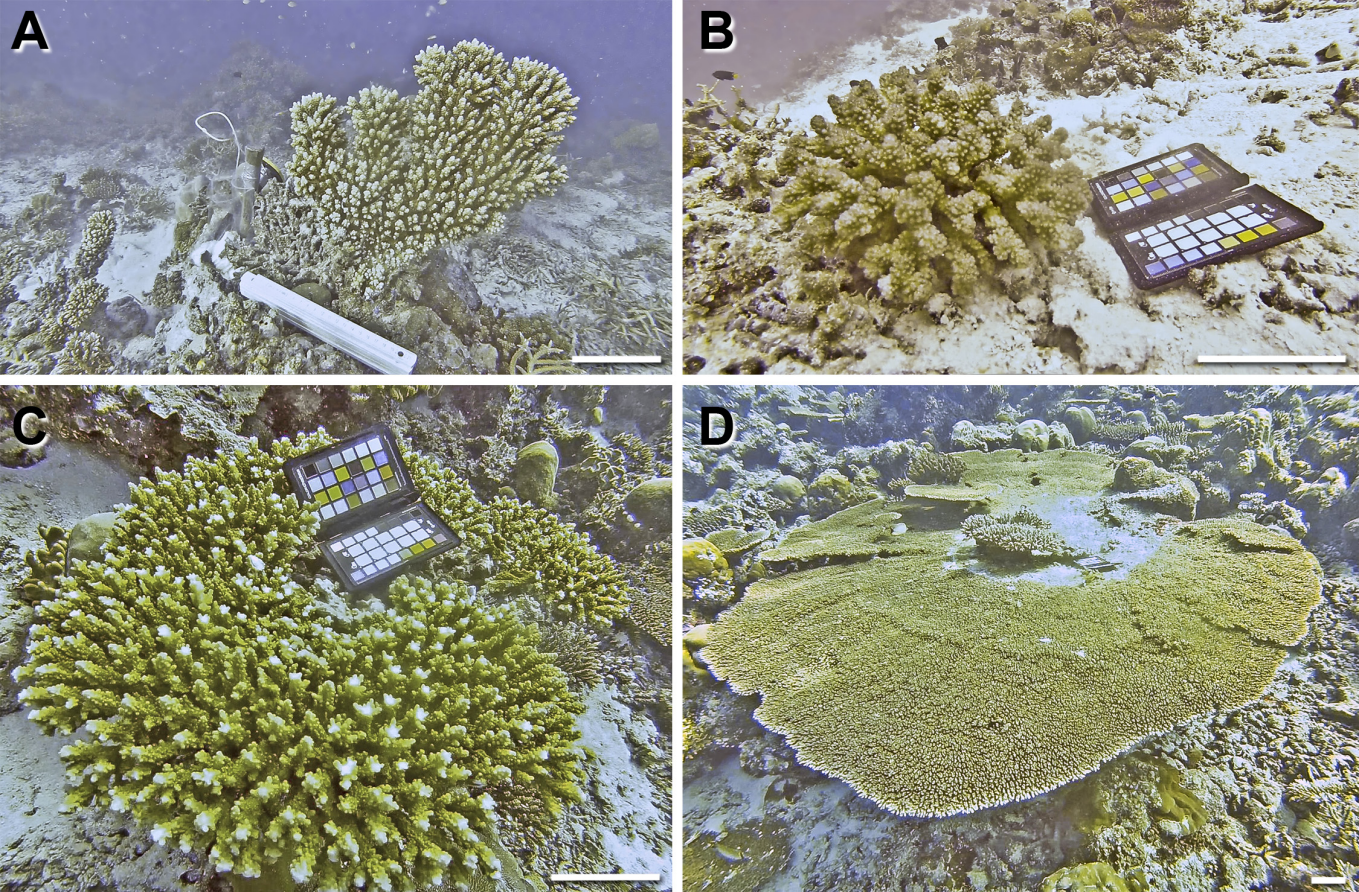
Branched and tabular coral growth forms sampled in this study (specie, location, date sampled). A) *Acropora* sp. 1, Home Reef, 08/05/14 B) *Pocillopora verrucosa*, Maaga, 17/05/14 C) *Acropora* sp. 2 (cf *valida*), Darambodhoo, 12/05/14 D) *Acropora hyacinthus* (tabular) Darambodhoo, 12/05/14. Scale bar 10 cm. Note: The coloured chart in some of the images is the ColorChecker Passport used for colour calibration and scale reference.

**Fig 4:**
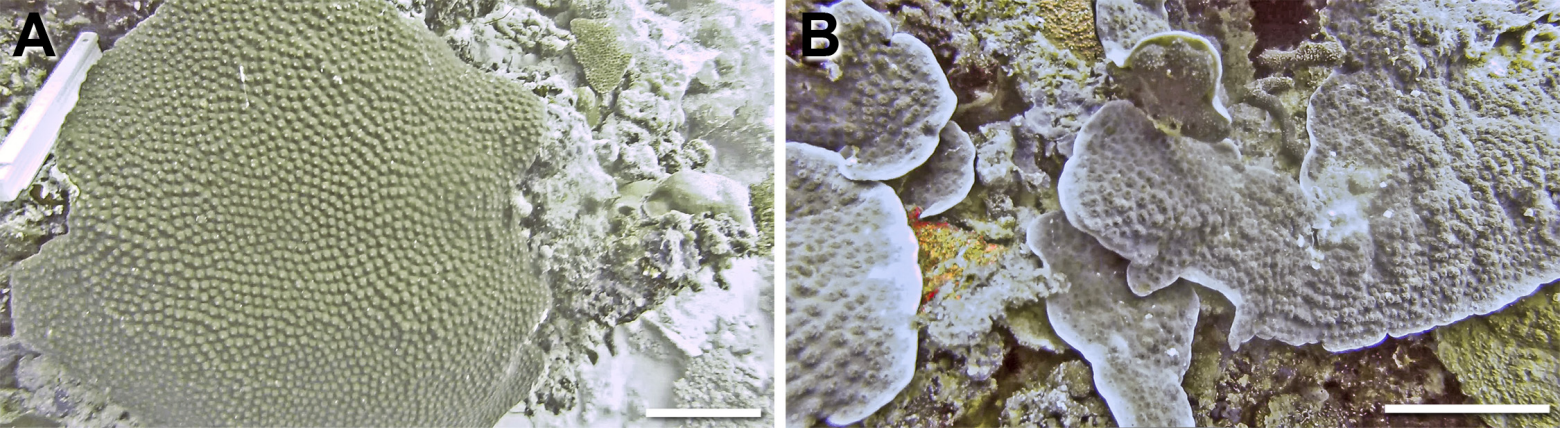
Encrusting and foliose coral growth forms sampled in this study (specie, location, date sampled). A) *Diploastrea heliopora* (encrusting), Home Reef, 08/05/14 B) *Echinopora lamellosa* (foliose), Wall Street, 17/05/14. Scale bar 10 cm.

A total of 10 coral colonies from 10 species and 5 growth forms were imaged *in situ* and sampled for 3D modelling and SA and V calculations The growth forms consisted of 4 massive corals (*Gardineroseris planulata*, *Porites lutea*, *P*. *lobata* and *Platygyra daedalea*) (Fig 2), 3 branched corals (*Acropora* sp. 1, *Acropora* sp. 2, *Pocillopora damicornis*) and 1 branched tabular coral (*Acropora hyacinthus*) (Fig 3), 1 encrusting coral (*Diploastrea heliopora*) (Fig 4A) and 1 foliose coral *Echinopora lamellosa*) (Fig 4B). Dr. Francesca Benzoni identified these corals on site.

**Fig 5:**
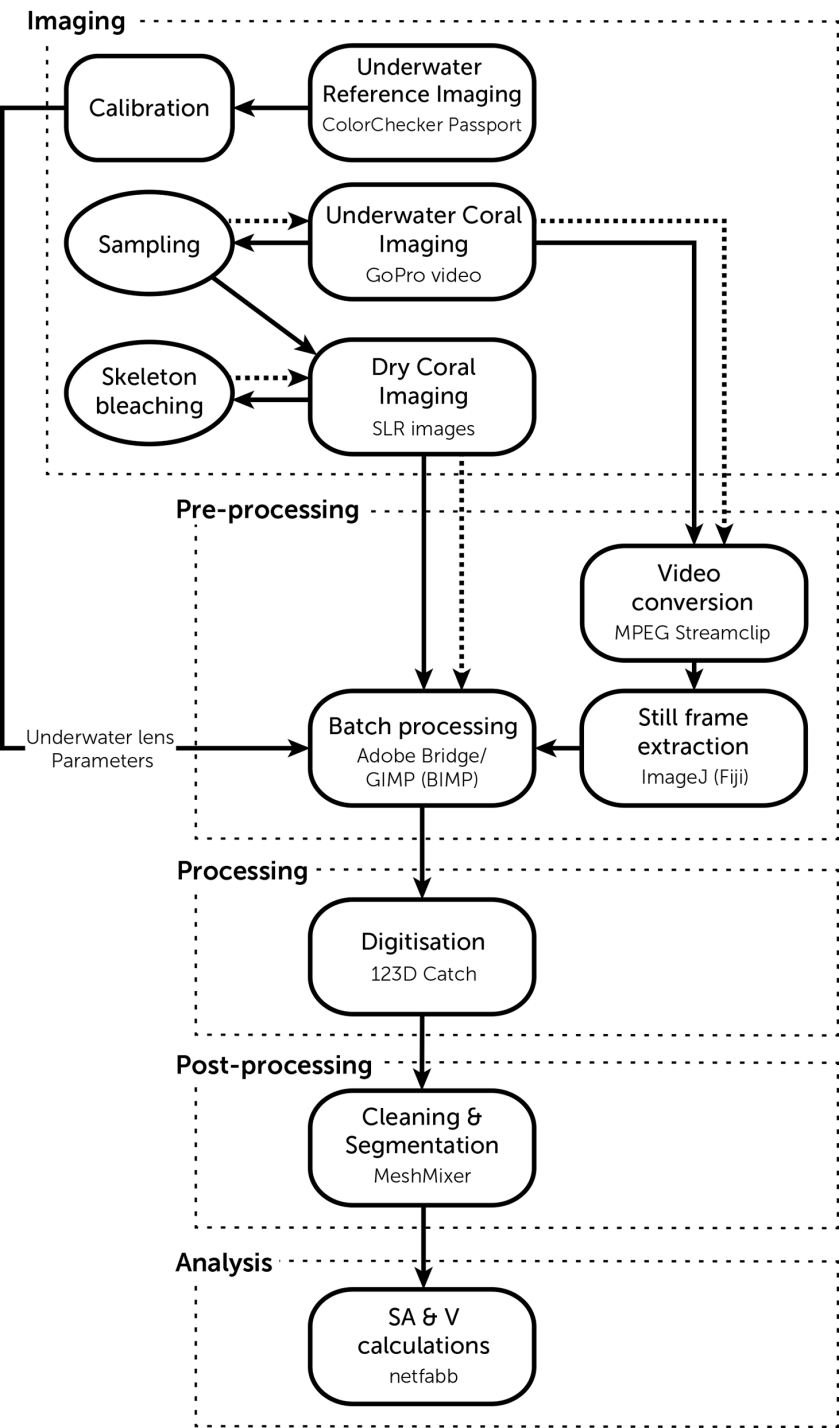
Digisitation pipeline. Dotted boxes represent the steps of this methodology, rounded boxes represent digital actions, ovals represent physical actions, line arrows represent the flow of actions and dotted arrows represent reiterations (repeating underwater imaging after sampling and repeating dry imaging after bleaching skeletons).

A pipeline of our digitisation process can be observed in Fig 5.

### Underwater Imaging

Underwater imaging was performed using a GoPro Hero 2 HD camera (California, U.S.A.) (≈ 400 EURO) coupled with the Flat Lens dive housing (≈ 100 EURO) on HD setting in an MPEG-4 Part 14 file (mp4). This setup of camera was selected due to its ample versatility, availability, ease of use and low cost (≈ 500 EURO). A 23 cm white folding ruler with black markings or an open ColorChecker Passport (X-Rite) (≈ 100 EURO) was placed in close proximity to the coral colony being imaged for color balance correction and scale reference. Colonies were imaged *in situ* by video recording two sets of full circles taken around the target colony, in a slightly different trajectory (to provide more camera positions for 3D reconstruction and reduce occlusions) and making sure the edges and the different perspectives of the colony are imaged. Then, a small portion of the colony was sampled using a hammer and a chisel and the colony was imaged again (to generate 'Sampled' 3D models to compare to corresponding 'Unsampled' 3D models) in the same manner.

### Dry Imaging

Collected coral colony samples were promptly placed in a tank with seawater and taken out for dry imaging on land. Each specimen was placed next to a high contrast ruler on a waterproof surface with a heavily textured design consisting in a different colour palette. Imaging was performed in an outdoor setting with a slightly overcast sky or in an indoor setting inside a clear coloured room with 4 fluorescent bulbs placed evenly, to reduce harsh lights and shadows. Some branched specimens were placed in a polystyrene mosaic to keep them upright, except for *Acropora* 1.

Three sets of photographs were taken full circle around the specimens, as described by Gutierrez-Heredia (2015) [34] with a Canon 40D SLR camera with a Canon Ultrasonic EF 100mm f/2.8 Macro USM lens on a Monami tripod using TTL metering exposure with a small aperture (f/10-f/11) in JPEG format in 2816 × 1880 resolution. The first set included an encircling ground view around the specimen with several overlapping images. The second set involved a similar imaging pattern of the previous set, taken at a higher angle (≈ 45°). A smaller camera separation (hence an increase in the number of pictures) was required to accurately image more complex specimens. The third set included macro images of individual corallites taken in a similar manner focusing and zooming in on a specific corallite or region of the specimen for macro imaging. After imaging, samples were bleached overnight in a 6% sodium hypochlorite solution (household bleach), dried and imaged as previously described.

### Image Preprocessing

An Intel Quad Core 2.8 GHz desktop computer with 64 GB RAM under Windows 7 was used for preprocessing and processing of the underwater videos and images in a lab. This piece of equipment was used due to availability, though it can also be performed with an off-the-shelf laptop. Videos were exported to Audio Video Interleave (AVI) files with the freeware MPEG Streamclip 1.2 (Squared 5) as Apple photos at 100% quality and no sound. The converted video files were opened in the open source package Fiji v2.0.00-rc-9 (ImageJ) and JPEG images were exported from still frames where the coral colony was well defined (sharp). An average of 58 (36–73) images were produced for the whole *in situ* colonies set and 71 (45–82) for the colonies after sampling. Both the underwater and the dry imaging sets were exported to Adobe Bridge CS5 (Adobe Systems, U.S.A.) for batch pre-processing, to enhance contrast, adjust light curves and exposure, sharpen features, reduce noise and correct lens distortion for calibration.

### Image Processing

Image sets were then processed using the downloadable version of 3D modelling and design software 123D Catch v3.0.0.54 (Autodesk Inc., San Rafael, CA, U.S.A.) for Windows, which was also used to align, crop, register and establish scale references using epipolar lines by means of the imaged reference object (ruler or ColorChecker Passport), using at least 15 reference images for each landmark for accuracy. This software uses automatic feature recognition and semi-automatic 3D model generation if manual points are placed when a generated model contains misalignments. The 3D models were exported with maximum quality mesh as a Wavefront object (OBJ). Models were imported into Windows Meshmixer 2.6 (Autodesk) for mesh cleaning, hole repair (flat remeshed) for Volume (V) and segmentation. 3D models from unsampled and sampled colonies were imported and aligned to each other, so segmentation would correspond as closely as possible, and exported as.OBJ. Subsequently, 3D models of coral colonies and samples were segmented from the rest of the 3D environment specifically for Effective Surface Area (ESA) calculations and exported.

### 3D measurements and analyses

The software Netfabb Studio Basic (NETFABB) (v 4.9.3) for Apple was used to calculate resulting Surface Area (SA) and Volume (V) analysis with the standard analysis tool for each 3D model. All these calculations are considered to be performed digitally on 3D models, except for water volume calculations (TV Water). Table 1 shows the criteria used to calculate each parameter SA and V analysis.

**Table 1:** Detail of criteria used for digital analysis of Surface Area and Volume of 3D models.

Category	Criteria	Surface Area	Volume
Underwater	Unsampled	Complete 3D model of unsampled colony after segmentation from community	Complete 3D model of unsampled colony after segmentation from community
	TSA/TV Sampled	Complete 3D model of sampled colony after segmentation from community	Complete 3D model of sampled colony after segmentation from community
	ESA Sampled	3D model of sampled colony after segmentation of non-living contact areas	Not applicable
Dry	TSA/TV Tissue	Complete 3D model of fresh sample after segmentation from imaging stage	Complete 3D model of fresh sample after segmentation from imaging stage
	ESA Tissue	3D model of fresh sample after segmentation of non-living contact areas	Not applicable
	TSA/TV Skeleton	Complete 3D model of skeleton sample after segmentation from imaging stage	Complete 3D model of skeleton sample after segmentation from imaging stage
	ESA Skeleton	3D model of skeleton sample after segmentation of non-living contact areas	Not applicable
	TV Water	Not applicable	Immersion of skeleton sample in water
Analysis	ThSA/ThV	Unsampled SA minus ESA Sampled	Unsampled V minus TV Sampled
	Biosurface/Biomass	TSA Tissue minus TSA Skeleton	TV Tissue minus TV Skeleton
	% Scale	% of ESA Tissue compared to Unsampled	% of TV Tissue compared to Unsampled
Errors	Theoretical Error	Calculated error of ThSA compared to ESA Tissue	Calculated error of ThV compared to TV Tissue
	Error TVW	Not applicable	% of TV Water compared to TV Skeleton

TSA (Total Surface Area), TV (Total Volume), ESA (Effective Surface Area), ThSA (Theoretic Surface Area), ThV (Theoretic Volume), Error TVW (Total Volume Water).

Effective Surface Area (ESA) is equivalent to Live Coral Surface Area [28], or “primary surface area”, which includes the SA of coral tissue and polyps [23]. The closer Theoretical Surface Area (ThSA) is to ESA Tissue, the accuracy of 3D digitisation and SA calculation are higher.

TV Water, or ‘real’ volume, was calculated on bleached skeleton samples using the laboratory standard technique of water displacement, which represents the most accurate method for volume measurement [27].

## Results

### 3D digitisation pipeline

**Fig 6:**
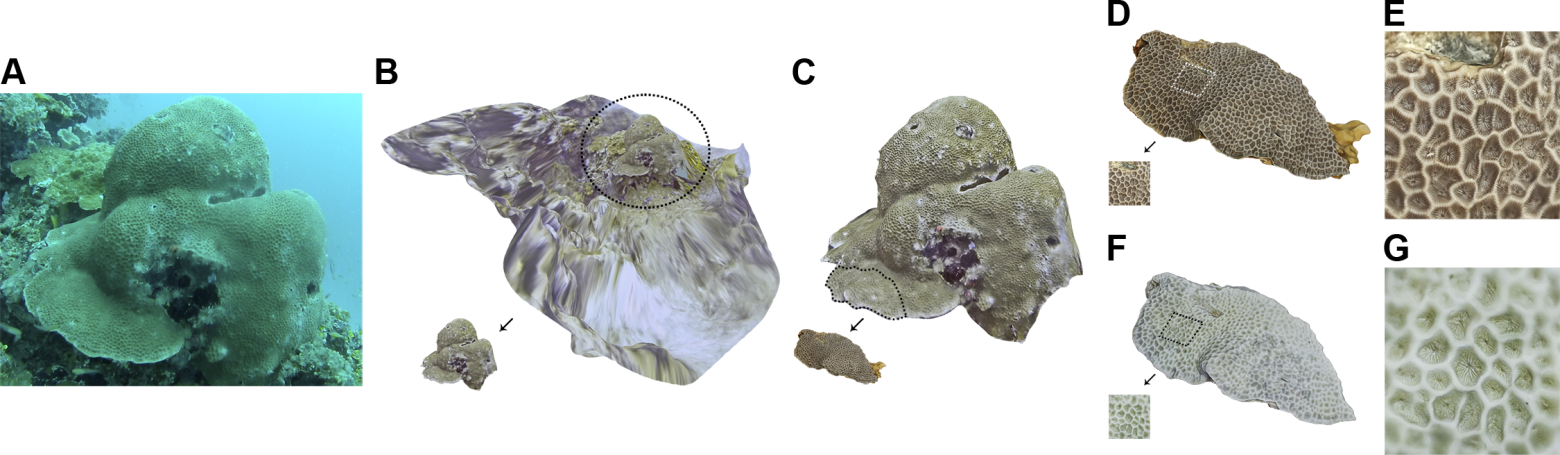
Digitisation pipeline of 3D coral sets. A) Unprocessed digital photograph of underwater *Gardineroseris planulata* colony extracted from video and used in model generation. B) 3D model at community scale of same underwater set where *G*. *planulata* colony was segmented from. C) 3D model at colony scale showing extraction of sample from *G*. *planulata* colony. D) 3D model at subcolony level of *G*. *planulata* using dry imaging on sample with live tissue displaying section where detailed macro imaging was performed. E) Detail of 3D macro imaging of live tissue sample of *G*. *planulata*. F) 3D model at subcolony level of *G*. *planulata* using dry imaging on same sample of bleached skeleton displaying section where detailed macro imaging was performed. G) Detail of 3D macro imaging of bleached skeleton sample of *G*. *planulata*.

10 sets of high definition and high resolution 3D models of coral specimens with different morphologies and imaged at different scales (community m's, colony, sub-colony and corallite) and modalities (underwater, dry, living tissue and bleached skeleton) were generated (Fig 6). An uncomplicated workflow for generating these sets was created so these techniques can be conveniently taught to non-specialists in coral research or computer science, so there is the possibility that these datasets can be generated for surveying and monitoring on a larger scale (ms to 100's of ms).

### 3D model generation

The amount of images, video time and total disk space used for generating 3D models from underwater coral colonies and their samples is represented in Table 2.

**Table 2:** Digital specifications in coral imaging.

Media	Coral	Per imaging set	Post-processing	Total disk space
Dry imaging	Fresh samples	58 images (39–85)	≈100 images	≈230 Mb
	Skeleton samples	62 images (45–85)	≈120 images	≈260 Mb
Videos	Underwater	30 secs–2 mins	≈ 200 images	≈570 Mb

Figures are averages and between parentheses, lower and upper counts. Amount of images used for macro photography is included in these figures.

**Fig 7:**
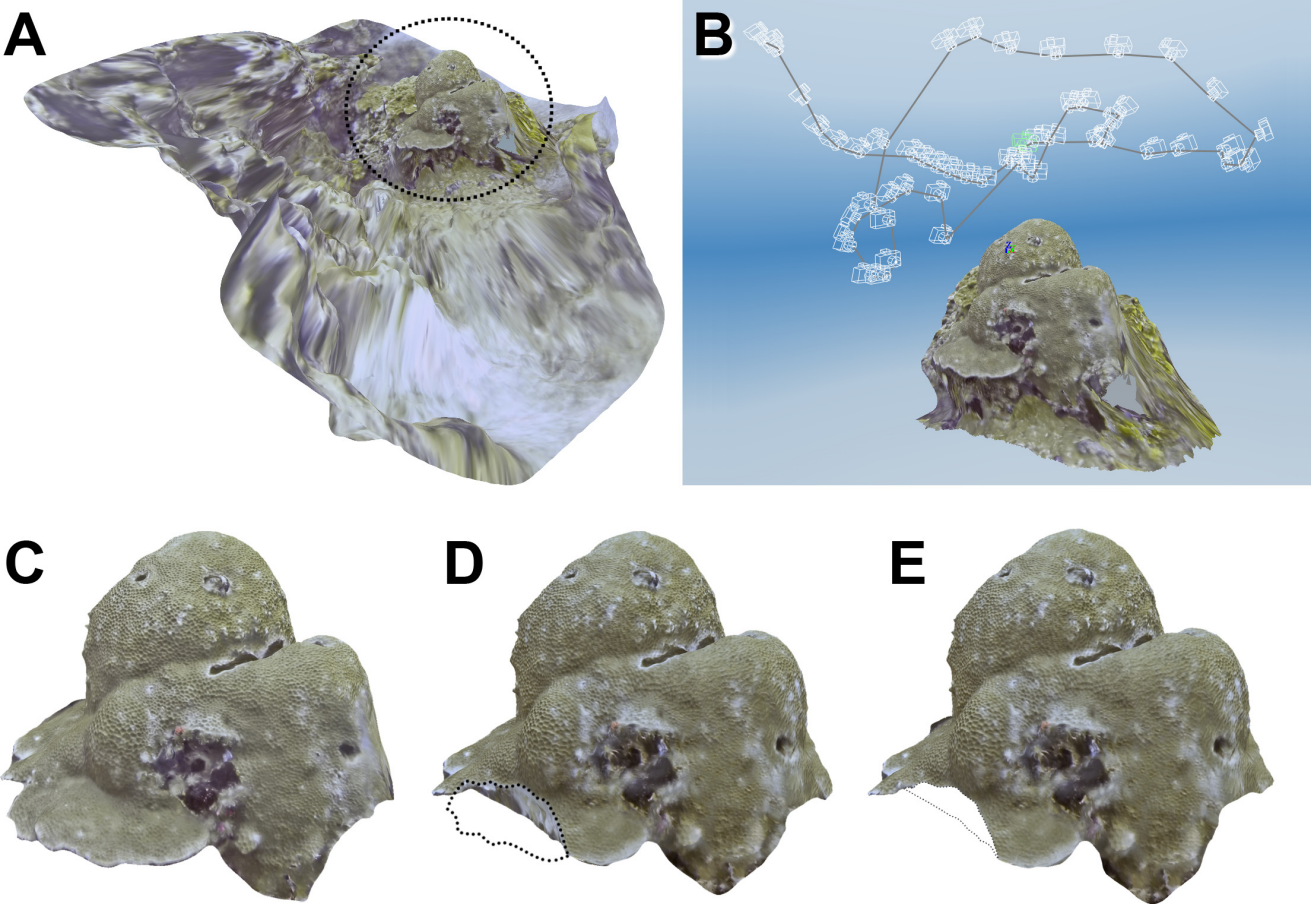
Multi-image photogrammetry of underwater coral colonies and segmentation. A) 3D model of community around *Gardineroseris planulata* colony. B) Set of camera points after alignment. C) Segmentation of colony for Unsampled calculations. D) Segmentation of colony for TSA and TV Sampled calculations. E) Segmentation of colony for ESA “Sampled” calculations. Dashed line represents area or outline of segmented portion of 3D model.

**Fig 8:**
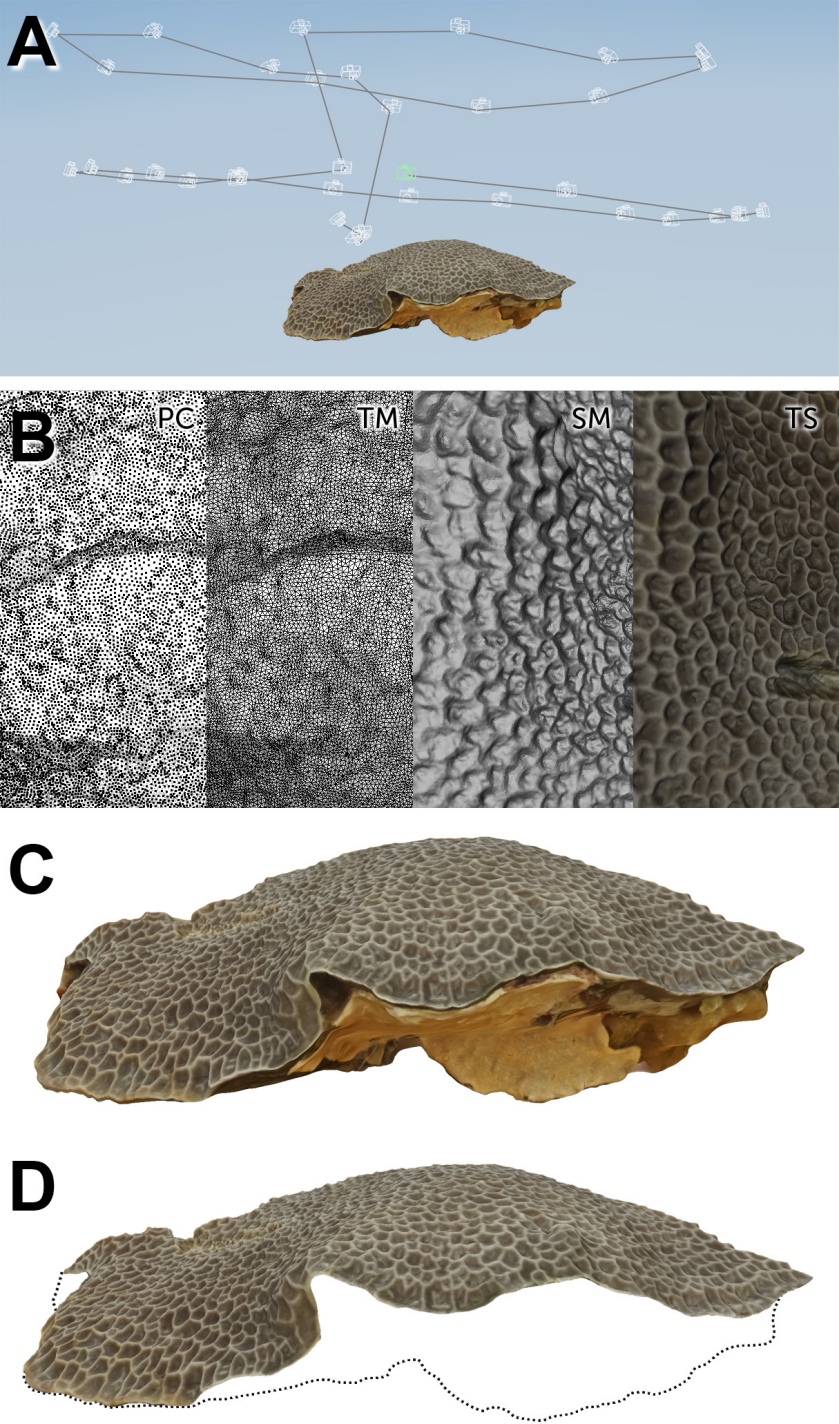
Dry multi-image photogrammetry of coral samples and segmentation. A) Set of camera points after alignment of *Gardineroseris planulata* imaging sample. B) PC = 3D point cloud. TM = Triangle mesh. SM = Untextured surface mesh. TS = Textured and refined surface mesh. C) Segmentation of 3D sample for TSA and TV Tissue and Skeleton calculations. D) Segmentation of 3D sample for ESA Tissue and Skeleton calculations. Dashed line represents outline of segmented portion of 3D model.

The resulting 3D models of underwater colonies and dry samples were similarly constructed due to the identical processing they were submitted to (Fig 7 and Fig 8).

### Detailed corallite imaging

**Fig 9:**
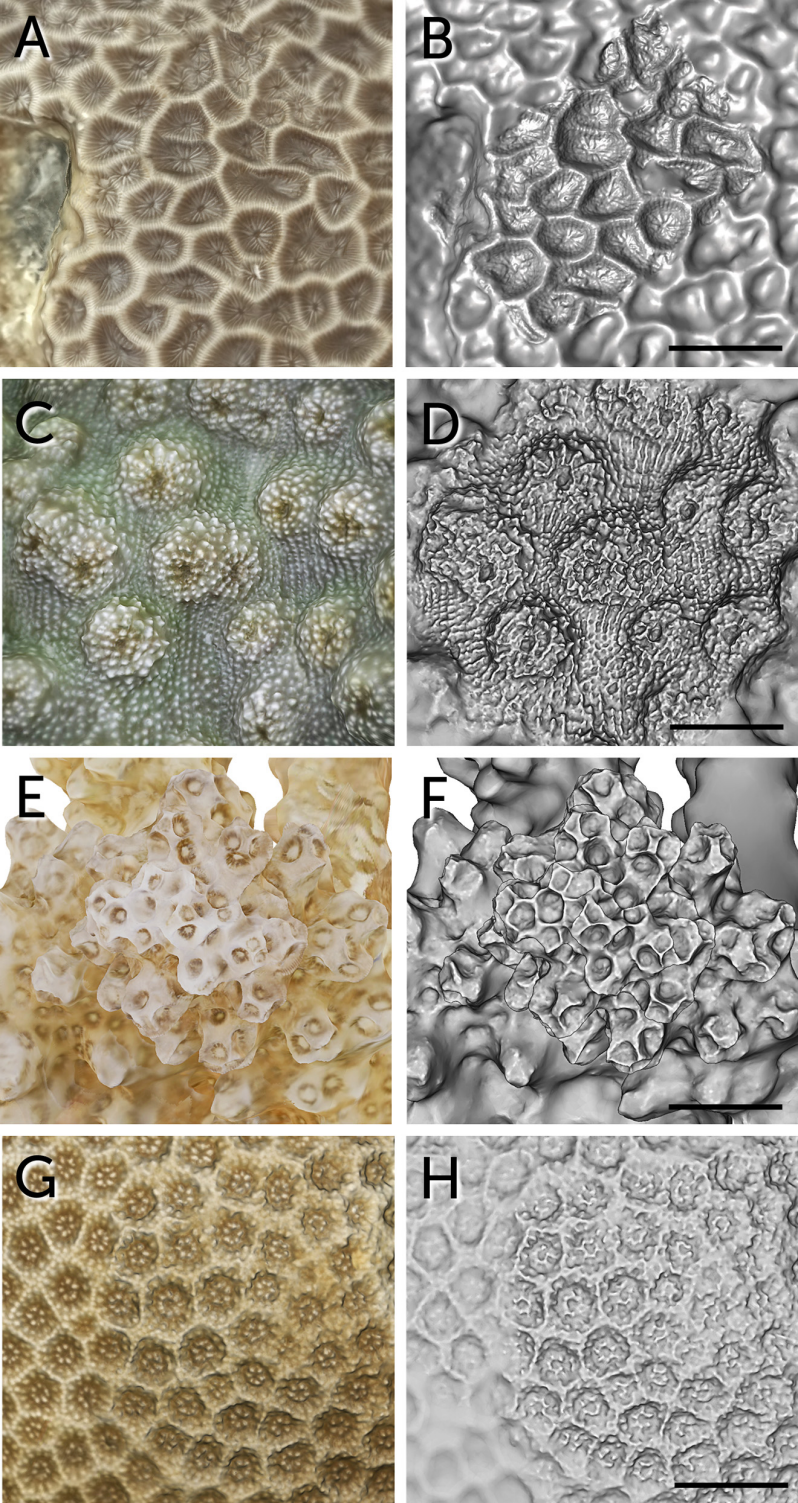
Macro detail of 3D models for taxonomical imaging. A) Textured 3D surface mesh of *Gardineroseris planulata*. B) Same untextured 3D surface mesh of *Gardineroseris planulata*. Scale bar represents 7.6 mm. C) Textured 3D surface mesh of *Echinopora lamellosa*. D) Same untextured 3D surface mesh of *Echinopora lamellosa*. Scale bar represents 6.8 mm. E) Textured 3D surface mesh of *Pocillopora verrucosa*. F) Same untextured 3D surface mesh of *Pocillopora verrucosa*. Scale bar represents 4.4 mm. G) Textured 3D surface mesh of *Porites lutea*. H) Same untextured 3D surface mesh of *Porites lutea*. Scale bar represents 3.6 mm.

In most of the 3D models of underwater colonies and dry samples a section of the digital mesh consisted of a region with a denser 3D point count (Fig 9). This is the area where macro imaging was performed and a finer detail of corallites was observed. In the species with bigger corallites, like *G*. *planulata*, the texture mapping and surface detail was detailed enough to provide adequate morphological observations suitable for taxonomic studies. A SA and V calculation for each corallite in a region could potentially be achieved. For species with a smaller size of corallites, calculations like corallite density per cm^3^ could be performed as well.

### Surface Area

The tabular coral morphology (*A*. *hyacynth**u**s*) has the largest colony SA, followed by massive species. Tabular and branched morphologies result in the largest SA from dry imaging. Tabular and branched specimens also produce dry imaging 3D models with the highest Biosurface measurements. The highest Theoretical Errors in SA are observed in the largest massive species, followed by the tabular morphology. Across morphologies, Unsampled SA is congruent with both Sampled TSA and ESA, which show a degree of precision between imaging models, especially when % Scale is lower (Table 3). Underwater SA shows a consistent decrease from Unsampled to Sampled calculations, except for *P*. *lutea* (Table 3 - ThSA). Additionally, SA calculations between Tissue and the Skeleton samples show this same consistency, except for *P*. *daedalea* and *E*. *lamellosa* (Table 3 - Biosurface). Biosurface in *P*. *daedalea* and *P*. *lobata* is virtually negligible. There is a high discrepancy between ESA Tissue calculations and ThSA, and there is no clear proportional pattern related to % Scale. The 3D models with the least Theoretical Error are *G*. *planulata* (9.16%) and *E*. *lamellosa* (15.27%). Precision in underwater SA tends to increase as TSA of the organism increases and % Scale decreases.

**Table 3:** Surface area measurements from 3D models of corals generated by underwater and dry multi-image photogrammetry.

Surface area (cm^2^)		Underwater			Dry				Analysis			Errors
Growth form	Corals	Unsampled	TSA Sampled	ESA Sampled	TSA Tissue	ESA Tissue	TSA Skeleton	ESA Skeleton	ThSA	Biosurface	% Scale	Theoretical Error
Massive	*Platygyra daedalea*	381.50	394.58	351.33	93.22	**47.26**	93.68	48.39	30.17	-0.46	12.39	36.17
	*Gardineroseris planulata*	2,703.69	2,587.70	2,560.16	410.80	**131.49**	353.48	118.07	143.53	57.32	4.86	9.16
	*Porites lutea*	9,821.73	9,982.85	9,927.23	153.12	**89.41**	150.17	87.45	-105.50	2.95	0.91	218.00
	*Porites lobata*	18,026.33	17,911.17	17,874.44	82.90	**44.80**	82.85	43.99	151.89	0.05	0.25	239.07
Branched	*Pocillopora verrucosa*	874.50	816.93	744.30	347.13	**344.93**	285.63	269.00	130.20	61.50	39.44	62.25
	*Acropora 1*	1,913.70	1,715.42	1,678.65	451.17	**419.56**	403.30	379.53	235.05	47.87	21.92	43.98
	*Acropora 2*	3,196.79	3,008.23	2,942.44	158.21	**151.87**	137.31	130.34	254.35	20.90	4.75	67.48
Tabular	*Acropora hyacynthus*	46,686.73	45,673.30	45,673.30	379.47	**372.89**	304.47	298.31	1,013.43	75.00	0.80	171.78
Crustose	*Echinopora lamellosa*	1,350.82	1,246.37	1,246.37	191.17	**90.62**	195.32	95.11	104.45	-4.15	6.71	15.27
	*Diploastrea heliopora*	2,685.03	2,658.66	2,577.36	293.61	**52.91**	271.47	48.90	107.67	22.14	1.97	103.48

TSA (Total Surface Area), ESA (Effective Surface Area), ThSA (Theoretic Surface Area). Surface Area calculations in bold should theoretically be analogous.

### Volume

Massive morphologies resulted in the highest colony V calculations. This morphology also produced high V measurements from dry imaging, followed by branched species. There is no conspicuous trend in Biomass calculations corresponding particular morphologies. The highest Theoretical Errors were observed in branched specimens, followed by massive morphologies. The highest Errors in TVW were observed in tabular and branched specimens. Across morphologies, a decrease in volume is observed between Unsampled TV and sampled TV (Table 4). Likewise, Tissue TV has a tendency to be consistent with TV Skeleton, and as can be observed in Biomass, reduction in volume is constantly observed except in *P*. *lobata* and *P*. *verrucosa* cases by a small amount, and it is practically negligible in *Acropora* 2 (Table 4 –Biomass). TV Tissue appears to be very little congruent with ThV, as can be seen with high estimates of Theoretical Error. Theoretical Error V of *P*. *daedalea* is the most accurate calculation with 9.4% of error. It seems Theoretical Errors directly increase with the TV of the coral colony, and also are indirectly related to the % Scale size of sample to colony. There is a large discrepancy between TV skeleton and TV water, as can be observed in Error TVW (Table 4). The smaller volume error between bleached skeletons and water displacement volume calculation is the Error TVW of *E*. *lamellosa* (6.34%). Some volume errors for bleached and volume displacement are large as close to 300% (*A*. *hyacinthus*). A constant underestimation of volume by TV Water (water displacement) is observed in relation to TV Skeleton. (Table 4 –Error TVW). Precision in underwater calculations also tends to increase as TV of the organism is larger, similarly to SA. Underwater TV could not be calculated for *A*. *hyacinthus* and *E*. *lamellosa*.

**Table 4:** Volume measurements from 3D models of corals generated by underwater and dry multi-image photogrammetry.

Volume (cm^3^)		Underwater		Dry			Analysis			Errors	
Growth form	Corals	Unsampled	TV Sampled	TV Tissue	TV Skeleton	TV Water	ThV	Biomass	% Scale V	Theoretical Error	Error TVW
Massive	*Platygyra daedalea*	664.18	616.28	**43.79**	*40*.*39*	*27*	**47.90**	3.39	6.59	9.40	49.61
	*Gardineroseris planulata*	12,978.50	12,742.50	**365.99**	*314*.*78*	*240*	**236.00**	51.21	2.82	35.52	31.16
	*Porites lutea*	101,661.40	100,402.00	**129.62**	*126*.*91*	*84*	**1,259.40**	2.71	0.13	871.64	51.08
	*Porites lobata*	147,841.20	146,957.60	**36.22**	*37*.*30*	*28*	**883.60**	-1.08	0.02	2,339.54	33.23
Branched	*Pocillopora verrucosa*	1,706.10	1,198.70	**69.03**	*73*.*69*	*55*	**507.40**	-4.66	4.05	635.03	33.98
	*Acropora 1*	3,138.20	2,610.90	**250.04**	*217*.*41*	*67*	**527.30**	32.63	7.97	110.89	224.49
	*Acropora 2*	6,008.30	5,075.40	**30.37**	*30*.*06*	*19*	**932.90**	0.31	0.51	2,971.52	58.22
Tabular	*Acropora hyacynthus*	-	-	**84.72**	*83*.*26*	*21*	**-**	1.46	-	-	296.46
Crustose	*Echinopora lamellosa*	-	-	**32.52**	*30*.*91*	*33*	**-**	1.61	-	-	6.34
	*Diploastrea heliopora*	4,571.90	3,980.60	**179.47**	*174*.*46*	*127*	**591.30**	5.01	3.93	229.47	37.37

TV (Total Volume), ThSA (Theoretic Volume), Error WV (Error to Total Volume Water). Volume calculations in bold and italics should theoretically be analogous respectively.

### Time

Imaging of underwater colonies was performed in a relatively short amount of time, depending on the size of the colony (Table 5). Most of the time was spent extracting still images from the video files (pre-processing) and particularly generating the 3D models (processing) (Table 5 –Underwater colonies). The average time needed end-to-end for an underwater 3D model was 9 hours, though it can take less if alignment is effective on the first attempts. On the other hand, it can take up to 12 hours if alignment is incorrect. Imaging of dry samples necessitated an extensively longer amount of time than underwater colonies (Table 5 - Imaging). However, pre-processing and processing times were shorter (≈37% and ≈83%, respectively). Time needed for mesh cleaning and segmentation was similar. Generation of finished 3D models on average was 10% faster for dry samples than underwater colonies.

**Table 5:** Approximate time used to generate a 3D model and its area surface and volume calculations, and water volume estimation of bleached skeletons samples of each colony.

Time	Underwater colonies	Dry samples	Water volume
Imaging	1 min (30 sec– 2 min)	30 min (19 min– 43 min)	-
Pre-processing	30 min (27 min– 45 min)	11 min (8 min– 16 min)	-
Processing	6 hrs (3 hr– 11 hr)	5 hrs (4 hr– 8 hr)	-
Cleaning	25 min (15 min– 55 min)	29 min (14–40 min)	-
Measurements	1 min	1 min	15 mins (10 min– 20 min)
Total	9 hrs (5 hrs– 12 hrs)	8 hr (5 hrs– 9 hrs)	15 mins (10 min– 20 min)

Figures are averages across the different morphologies, and between parentheses are lower and upper approximations.

### Errors

**Fig 10:**
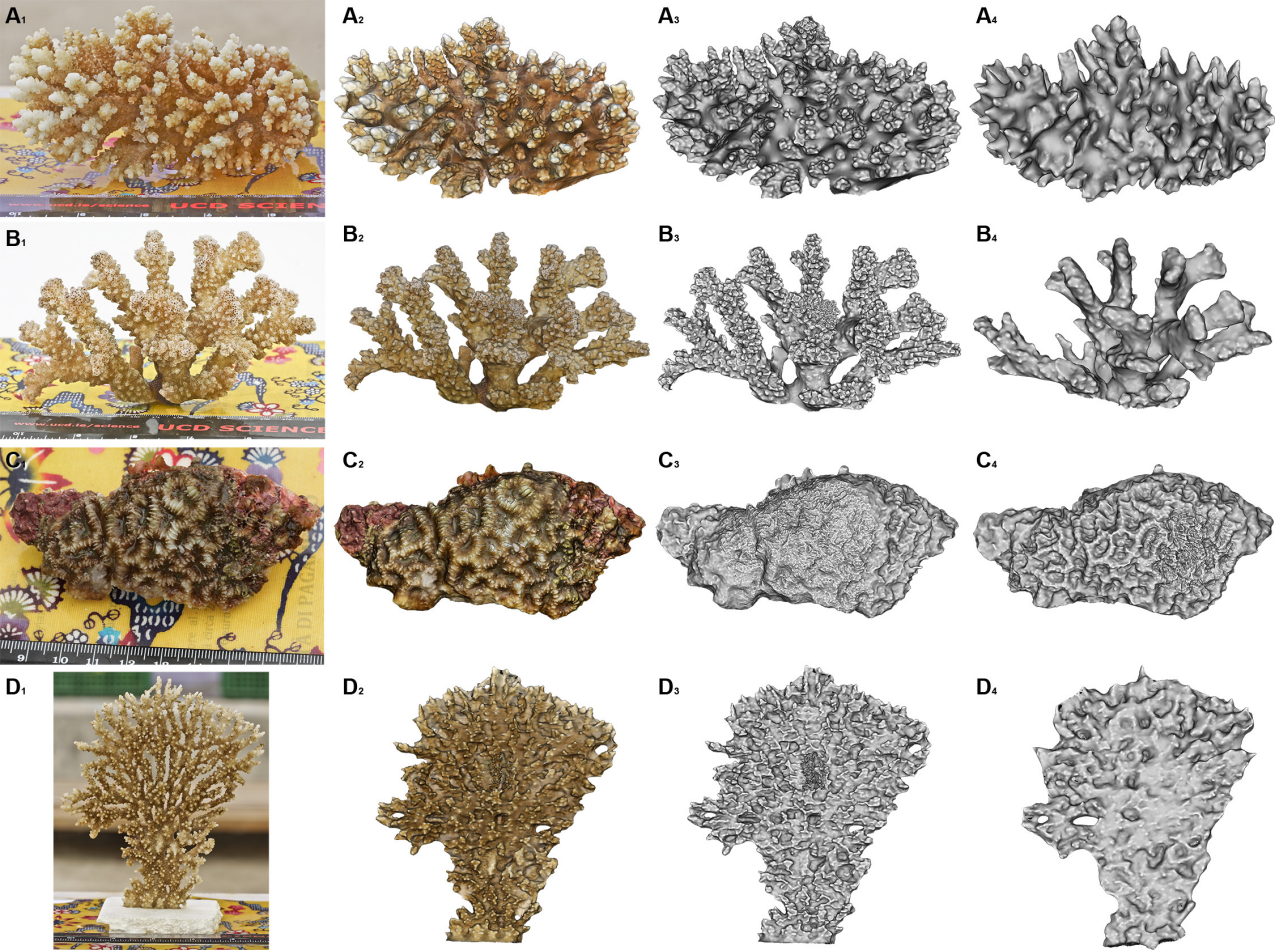
Over and underestimations in 3D models. A) *Acropora* sp. 1. B) *Pocillopora verrucosa*. C) *Platygyra daedalea*. D) *Acropora hyacinthus*. 1) Photographs from imaging session. 2) Texture mapped 3D model with shading of fresh sample. 3) Untextured 3D model of fresh sample. 4) Untextured 3D model of bleached skeleton.

There was an apparent difference in the 3D point cloud in models imaged with tissue and skeleton (Fig 10). This was more evident in branched specimens (Fig 10A and 10B–10D). The tips of the coral branches presented a higher point density than the central 'stalk' of the sample. This caused overestimations in SA and V from 3D model calculations. In the case of *P*. *daedalea* (Fig 10C), there is an evident layer of mucus across individual corallites from dry imaging 3D models. When the skeleton of *P*. *daedalea* was bleached, the detail of individual corallites was correctly digitised.

## Discussion

In this study existing methods of digital photogrammetry were combined to digitise 3D models of underwater colonies and dry samples at multiple scales using imaging from off-the-shelf monocular cameras. SA and V were quantified from colony to polyp scales, and high detailed map textures were generated for the 3D models which can aid in identification and health assessment studies. These techniques can be used to close the gap in knowledge of coral habitats and their health across scales increasing our capacity to assess and manage them.

One of the major problems observed with medium-range photogrammetry is noise, which can introduce an uncertainty between real and theoretical camera positions [25]. Turbidity and non-uniform lighting are the main factors producing this noise [27, 35]. GoPro seems to be proficient with imaging in bright conditions, but produces a considerable amount of noise when underexposed scenes and areas are pre-processed. This is further aggravated when JPEG or TIFF files are extracted from video footage. A solution to this would be the use of imaging equipment with RAW format output, which would also improve colour balance using colour calibration accessories such as the ColorChecker Passport. Sensor sizes don't seem to be a pressing factor in accuracy in 3D model generation [33]. Other options of off-the-shelf imaging equipment can still be used for this procedure, because the potential of non-metric cameras is still very high due to their versatility, light weight, low cost and other practical considerations [21, 33].

The “black box” characteristic of this particular software (123D Catch) can be either a detrimental quality to experts or an asset for monitoring to amateurs. The disadvantages of this software are low customization options both in processing procedures and point density of output product. This software also relies on internet speed and on online traffic capacity of its processing servers. This software has a limit of 70 images per 3D model generation as well. On the other hand, advantages of 123D Catch involve it being user-friendly, it is flexible with different scene parameters, there is a constant algorithm improvement [33] and having a graphic interface (especially versatile during alignment and scaling). 123D Catch handles an acceptable number of formats for output files and it does not require an extensively technical acquisition procedure, calibration methods, or extensive programming, mathematical or design knowledge. It runs on low RAM memory, disk space and processing power. Processing can be semi-automated if imaging is done correctly, and it offers an accommodating learning curve for new users. There are other open-source software bundles for photogrammetry based on Visual SFM [36] and CMPMVS [37]. This software bundle moves the processing done in online servers to the user's CPU or GPU, increasing the need of higher processing power, RAM memory and hard drive space.

An important knowledge gap that can be closed by photogrammetry is filling the current absence of a versatile tool useful for calculation of SA and V of complex 3D morphologies across different scales that can be used in taxonomic identification too. Provisional taxonomic identification using stereo-photogrammetry analysis [19] and rough taxonomical estimations through multi-image photogrammetry using SA calculations have been reported [28]. Additionally, further taxonomical identification has been demonstrated based on 3D models alone using photogrammetry [34]. This is essential for collaboration between researchers using the Internet and for creating online databases. This method has the possibility of calculating micro-scale 3D SA and V [38], and coral benthic assemblage rugosity and complexity [30, 31, 32] at the finer scales.

Certainly, the accuracy and limitations of the entire process is based on the quality and resolution of imaging [20, 26]. It is convenient to maintain intrinsic parameters constant and preserve this information during pre-processing so the 3D processing software can extract it as EXIF data from digital images. The digitising software was unable to extract EXIF data from images extracted from videos from underwater imaging and certainly affected accuracy and probably increased the amount of processing time. One of the advantages of this software is the on-the-fly calibration which can capture the calibration parameters needed for a particular subaquatic environment [11]. Using an image of an appropriate object (in this case the ColorChecker Passport) taken before video imaging can reduce the error during pre-processing produced by the automatic radial distortion correction used in 123D Catch [33].

Some of the inaccuracies in this paper's 3D models could be explained by fundamental errors introduced during alignment and scaling. Alignment is also affected by user error during manual alignment. In some 3D models, several attempts were uploaded for processing, specifically when small misalignments were detected when superimposing the output model with 2D images. A slight misalignment, particularly in larger colonies can extrapolate in higher inaccuracies during reconstruction, as can be observed in *P*. *lobata*, *P*. *lutea* and *A*. *hyacinthus*.

**Fig 11:**
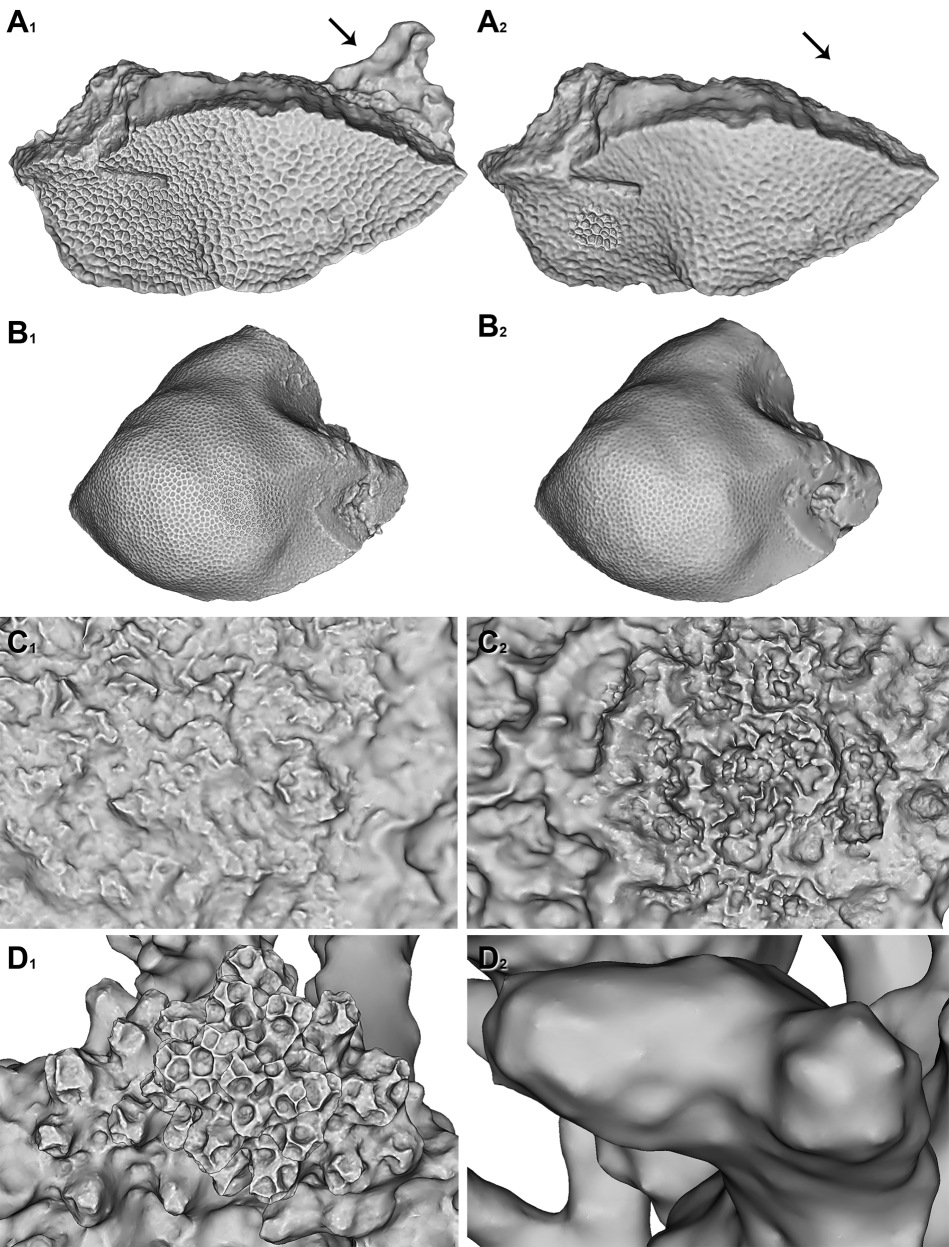
3D Models of fresh coral samples and bleached skeletons. A) *Gardineroseris planulata*. B) *Porites lutea*. C) *Platygyra daedalea*. D) *Pocillopora verrucosa*. 1) 3D Models with biomass. 2) Bleached skeleton 3D models. Black arrow represents location of sponge epiphyte before bleaching.

The consistent reduction of SA and V between underwater Unsampled and Sampled colonies, except in two cases for SA (*P*. *lutea* and *P*. *daedalea*), demonstrated that the technique was consistent and sensitive enough to differentiate changes while underwater. Nevertheless, this discrepancy related to V of coral samples is considerably larger than SA, particularly in *P*. *lobata* and *Acropora* 2 as can be observed in V Theoretical Error (Table 4). This error is proportionately larger as V % Scale is smaller. This indicates this method is less accurate concerning very fine changes in proportion of a larger colony size to a smaller size of samples. This sensitivity differentiating small changes between fresh tissue and the skeleton is also observed in SA and V calculations in dry imaging (Table 3 –Biosurface and Table 4 –Biomass). This is most notable in samples with a considerable amount of epiphytes sampled along with the colony fragment (Fig 11A, Table 3 –*G*. *planulata* Biosurface, and Table 4 –*G*. *planulata* Biomass), and viceversa in samples where only a thin Biosurface is present (Fig 11C, Table 3 –*P*. *lobata* and *P*. *lutea* Biosurface, and Table 4 –*P*. *lobata* Biomass). Negative values in Biosurface and Biomass in P. *daedalea* and *E*. *lamellosa* (Table 3 –Biosurface) can be addressed as a reduced SA calculation due to the presence of coral mucus in the cavities of corallites during dry imaging (Fig 11C), absent during dry imaging of its bleached skeleton. Macro dry imaging of coral samples also produced errors due to a high noise-to-signal ratio from bleached skeletons and reduced contrast of their features. This produced a much less detailed 3D model of coral skeletons than of fresh coral samples (Fig 11D) affecting calculations (Table 3 –*P*. *verrucosa* and *Acropora* spp. Biosurface). Additionally, isolated outer branches of species like *Acropora* sp. and *P*. *verrucosa* are well defined but the main central axis is depicted as a solid mass when occlusions and poor illumination in those crevices failed to accurately represent their complex topography (*Acropora* spp. and *P*. *verrucosa*, Fig 10A and 10B–10D). This certainly affected accuracy.

The high Theoretical Errors both in SA and V (Table 3 and Table 4) show that this digitisation method still needs improvement in accuracy when compared to other published results [22, 23, 26, 28, 33], particularly for underwater imaging. The dry imaging method has been demonstrated to be highly accurate [34]. It has to be remarked that these 3D sets can be produced using minimum off-the-shelf equipment and freeware (Adobe Bridge was used because of its availability, but the open source softwares ImageMagick from www.imagemagick.org/ or GIMP from https://www.gimp.org/ could equally be used for image edition and batch processing). The cost of equipment was approximately ≈ 500 EURO without considering the computer and storage equipment, internet charges and SCUBA diving. Adding more specialised equipment, accessories and/or software could improve accuracy and reduce processing time, with added cost. Another method to correct SA and V calculations to smaller discrepancies could use regression formulae [33]. Accuracy is an important asset to improve and consistency in precision is required for routine estimations of coral monitoring [26]. Due to topographic complexity of their colonies, *A*. *hyacinthus* and *E*. *lamellosa* didn't produce coherent solid 3D models suitable for V calculations (Table 4).

This photogrammetric dry macro imaging method has been demonstrated to be accurate both in SA and V calculations [21, 33, 34], particularly when compared to the water displacement volume of coral skeletons. In this study, digital V was constantly overestimated in relation to water volume. The same overestimation was previously reported [33]. This is affected by the differential water absorption due to inter and intraspecific porosity of scleractinian skeletons [39, 40]. A thin layer of paraffin or wrapping specimens in a thin film of plastic around the sample should be used when calculating water displacement accurately [22, 33].

The quantity of images used in other studies (4 to 20 images) [20, 22, 27, 41] is considerably lesser than the ones used in this study. These studies didn't produce 3D models with fine topography, especially at corallite level, their models didn't have a detailed texture map of the colony or sample, and didn't image corals more complex than massive or simple branches, though. One of the purposes of this paper is to generate 3D models with high quality morphology and texture mapping. Therefore, for this specific exhaustive approach a large number of images are required with sufficient orthogonal views in relation to planar surfaces of the colonies and samples. A high number of images used in the photogrammetric network also improves precision and reliability [20]. Up to 800 Mb of hard drive space (videos, pre-processed and processed images, 3D model files) can be needed to generate a 3D model set of a single colony, which could prove problematic if a reference collection of 100 specimens is needed for a project. However, processing speed increases, and cost and availability of hard drive space continues to drop as technology advances. Furthermore, by eliminating pre and post-processed images and unsegmented 3D models after obtaining final 3D models, the hard drive space used can be reduced to 200–400 Mb per colony (with all the models digitised from unsampled and sampled underwater colonies and dry imaging from fresh and skeleton samples). Another problem compensated with this amount of images is producing sufficient coverage of complex specimens due to occlusions and crevices [19], obstruction by surrounding organisms and epiphytes [15] and clear delineation of the colony limits for segmentation. A high number of images stay in storage as a permanent record [19, 22], and can be re-used for further measurement if needed, especially if different or novel algorithms can be used to create new 3D models to compare to previous ones. Accurate digitisation of crevices and by occlusions in heavily branched or complex specimens, or underexposure due to *in situ* environmental lighting can also be improved using external lights, (diffuse strobes). This will increase the cost of the setup, though.

One of the advantages readily observed by photogrammetry is the very high speed of *in situ* imaging, [12] coupled with the low time needed to place the reference object and extracting a sample of the colony (5 minutes) if needed. Processing and analysis work time is moved from the field to the lab, effectively decreasing the dangers of SCUBA diving [19] and reducing effort on the field. Authors employing photogrammetry inform 2 to 7 hrs in producing a 3D model [22, 26, 27]. 3–4 hours has been considered enough time to deter the use of this technique for most routine surveys [26], while others mentioned this is suitable for routine applications [27]. 9 hours in average to generate a high quality 3D model of a colony would prevent most researchers to use this technique routinely in coral monitoring. Most of the digitising time is spent in manual operations (extracting frames from videos, alignment, segmentation, etc). This could be greatly reduced by using semi-automatic and automatic procedures [42], both in imaging and processing.

**Fig 12:**
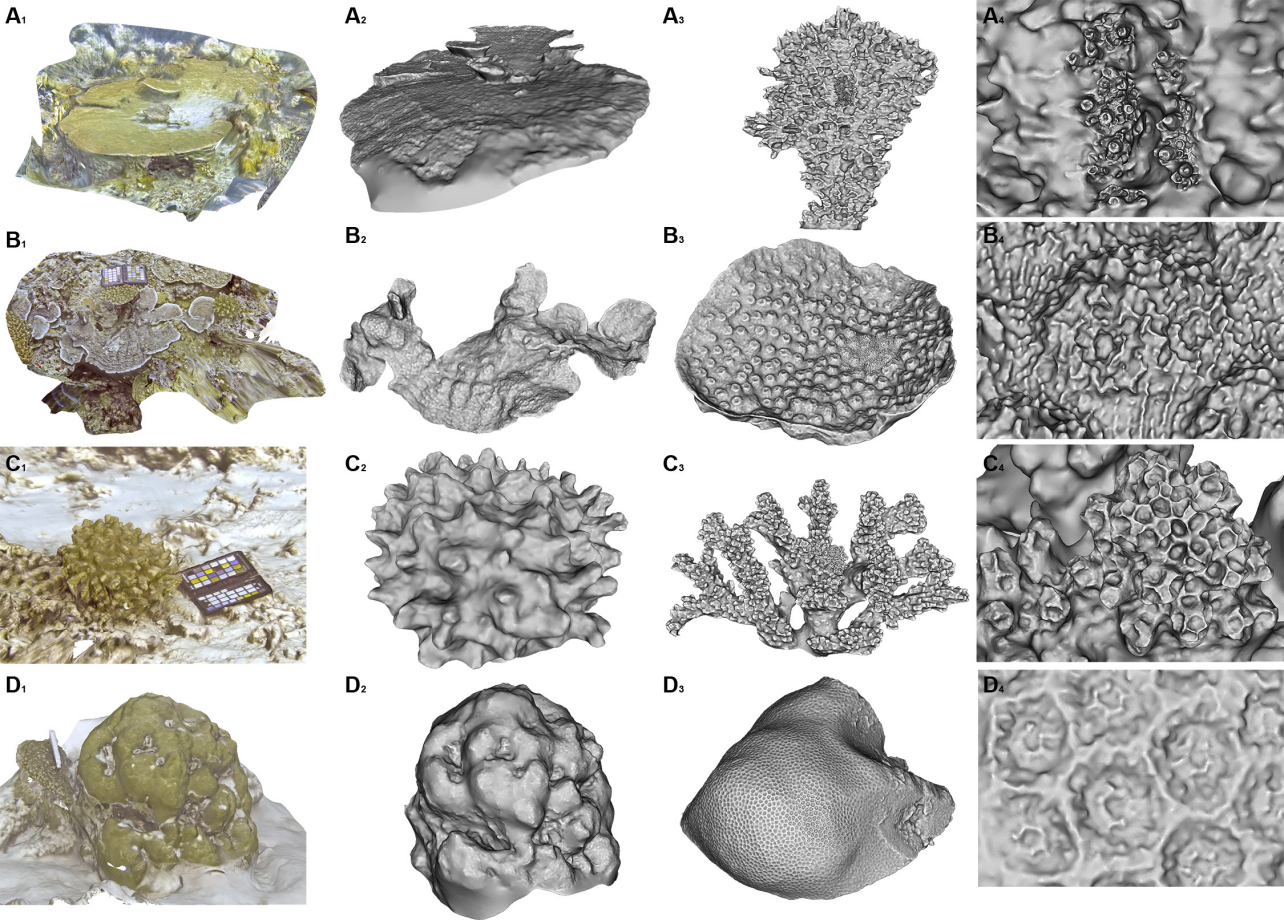
End-to-end 3D datasets. A) *Acropora hyacinthus*. B) *Echinopora lamellosa*. C) *Pocillopora verrucosa*. D) *Porites lutea*. 1) 3D environment with texture map around target colony. 2) 3D model of segmented colony. 3) 3D model of representative sample from colony. 4) Corallite detail in 3D model using close-range photogrammetry.

The most important aspect of this protocol at the moment is generating affordable high quality end-to-end multi-scale 3D models (Fig 12) that can be used to produce quantitative calculations across scales and can be readily uploaded online to share amongst collaborators. Scalability is a novel and important asset in coral studies [43]. These sets can be used as the base for online taxonomic digital libraries [34, 44], which can be complemented by other 3D imaging techniques, [45, 46] and readily accessible for experts or amateurs. 3D models can also be downloaded from the net and used in digital simulations [47], to display and share digitisations of valuable specimens of museum coral collections, and 3D printing for education.

## Conclusions

When undergoing biological, environmental and monitoring studies underwater, SCUBA divers must be well trained and experienced for visual estimates [20]. SCUBA divers require considerably more time and resources for more detailed estimations of coral assemblages, and their performance is not as efficient as on land, causing significant sampling bias and errors. Additionally, the number of experts in this field is severely reduced for the actual needs of coral research and monitoring, and training amateurs and laypersons can be a lengthy and costly enterprise. This is why a relatively fast, flexible, low-cost, non-invasive, user-friendly, portable, commercially available, and moderately impersonal system of measurements and recording is needed [20, 22, 23, 26]. This system must be based in available technology within limits imposed by the underwater environment and finite recourses of research groups. This photogrammetric workflow is promising, because a single diver using a low cost HD underwater camera and freeware produced these 3D sets. The relatively ease of this workflow also permits non-specialists to learn photogrammetry for coral monitoring, and that opens the possibility for local inhabitants to perform their own suveys and make accessible these datasets for coral researchers.

While accuracy and reconstruction time for photogrammetry still have room for improvement, other attributes of high quality 3D imaging can balance this disparity. These attributes are repeatability, multi-scaleability, generation of high quality texture mapping and complex topography, and the potential for multi-spectral 3D digitisation (UV). These 3D sets can be used for taxonomical, biodiversity, health assessment, and other morphometrical studies like rugosity and complexity. It is particularly useful for creating online dataset libraries for collaboration and education purposes, like digital display of museum collections. These 3D collections can also help fill in the gap in scaling up or down data sets and integrating measurements of related studies in coral research.

## Supporting Information

S1 File Statistics (OUTPUT.rtf). (RTF)

S2 File Tables and calculations for analysis (Calculations tables.xlsx). (XLSX)

S1 Video End-to-end digitisation and analysis of 3D models of *Gardineroseris planulata* from Wall Street dive site in the Maldives, from community to corallites. (https://vimeo.com/125794590).
(MOV)

S2 Video Echinopora lamellosa, 3D model of complex encrusting colony. (https://vimeo.com/109268768).
(MP4)
