# Supplementary material for: End to End Digitisation and Analysis of Three-Dimensional Coral Models, from Communities to Corallites
Source: PLoS One. 2016 Feb 22;11(2):e0149641. doi: 10.1371/journal.pone.0149641 (PMC4763093; doi:10.1371/journal.pone.0149641)
Supplement: S1 File — (RTF) [file pone.0149641.s001.rtf]

GRAPH
  /HISTOGRAM=VAR00003
  /PANEL ROWVAR=VAR00002 ROWOP=CROSS.


Graph


Notes	
Output Created	01-DEC-2015 14:55:28	
Comments		
Input	Active Dataset	DataSet0	
	Filter	<none>	
	Weight	<none>	
	Split File	<none>	
	N of Rows in Working Data File	20	
Syntax	GRAPH
  /HISTOGRAM=VAR00003
  /PANEL ROWVAR=VAR00002 ROWOP=CROSS.	
Resources	Processor Time	00:00:01.08	
	Elapsed Time	00:00:00.67	


[DataSet0] 


DESCRIPTIVES VARIABLES=VAR00003
  /STATISTICS=MEAN STDDEV MIN MAX.


Descriptives


Notes	
Output Created	01-DEC-2015 14:57:21	
Comments		
Input	Active Dataset	DataSet0	
	Filter	<none>	
	Weight	<none>	
	Split File	<none>	
	N of Rows in Working Data File	20	
Missing Value Handling	Definition of Missing	User defined missing values are treated as missing.	
	Cases Used	All non-missing data are used.	
Syntax	DESCRIPTIVES VARIABLES=VAR00003
  /STATISTICS=MEAN STDDEV MIN MAX.	
Resources	Processor Time	00:00:00.00	
	Elapsed Time	00:00:00.00	


[DataSet0] 


Descriptive Statistics	
	N	Minimum	Maximum	Mean	Std. Deviation	
VAR00003	20	351.33	46686.73	8660.8200	13884.87516	
Valid N (listwise)	20					

T-TEST PAIRS=VAR00003 WITH VAR00005 (PAIRED)
  /CRITERIA=CI(.9500)
  /MISSING=ANALYSIS.


T-Test


Notes	
Output Created	01-DEC-2015 15:00:23	
Comments		
Input	Active Dataset	DataSet0	
	Filter	<none>	
	Weight	<none>	
	Split File	<none>	
	N of Rows in Working Data File	20	
Missing Value Handling	Definition of Missing	User defined missing values are treated as missing.	
	Cases Used	Statistics for each analysis are based on the cases with no missing or out-of-range data for any variable in the analysis.	
Syntax	T-TEST PAIRS=VAR00003 WITH VAR00005 (PAIRED)
  /CRITERIA=CI(.9500)
  /MISSING=ANALYSIS.	
Resources	Processor Time	00:00:00.00	
	Elapsed Time	00:00:00.01	


[DataSet0] 


Paired Samples Statistics	
	Mean	N	Std. Deviation	Std. Error Mean	
Pair 1	VAR00003	8764.0820	10	14391.78170	4551.08098	
	VAR00005	8557.5580	10	14136.95598	4470.49801	


Paired Samples Correlations	
	N	Correlation	Sig.	
Pair 1	VAR00003 & VAR00005	10	1.000	.000	


Paired Samples Test	
	Paired Differences	
	Mean	Std. Deviation	Std. Error Mean	95% Confidence Interval of the Difference	
				Lower	
Pair 1	VAR00003 - VAR00005	206.52400	301.04037	95.19732	-8.82731	

Paired Samples Test	
	Paired Differences	t	df	Sig. (2-tailed)	
	95% Confidence Interval of the Difference				
	Upper				
Pair 1	VAR00003 - VAR00005	421.87531	2.169	9	.058	

T-TEST PAIRS=VAR00003 WITH VAR00005 (PAIRED)
  /CRITERIA=CI(.9500)
  /MISSING=ANALYSIS.


T-Test


Notes	
Output Created	01-DEC-2015 15:02:37	
Comments		
Input	Active Dataset	DataSet0	
	Filter	<none>	
	Weight	<none>	
	Split File	<none>	
	N of Rows in Working Data File	20	
Missing Value Handling	Definition of Missing	User defined missing values are treated as missing.	
	Cases Used	Statistics for each analysis are based on the cases with no missing or out-of-range data for any variable in the analysis.	
Syntax	T-TEST PAIRS=VAR00003 WITH VAR00005 (PAIRED)
  /CRITERIA=CI(.9500)
  /MISSING=ANALYSIS.	
Resources	Processor Time	00:00:00.00	
	Elapsed Time	00:00:00.01	


[DataSet0] 


Paired Samples Statistics	
	Mean	N	Std. Deviation	Std. Error Mean	
Pair 1	VAR00003	174.5740	10	146.39888	46.29539	
	VAR00005	8557.5580	10	14136.95598	4470.49801	


Paired Samples Correlations	
	N	Correlation	Sig.	
Pair 1	VAR00003 & VAR00005	10	.311	.382	


Paired Samples Test	
	Paired Differences	
	Mean	Std. Deviation	Std. Error Mean	95% Confidence Interval of the Difference	
				Lower	
Pair 1	VAR00003 - VAR00005	-8382.98400	14092.13055	4456.32296	-18463.88691	

Paired Samples Test	
	Paired Differences	t	df	Sig. (2-tailed)	
	95% Confidence Interval of the Difference				
	Upper				
Pair 1	VAR00003 - VAR00005	1697.91891	-1.881	9	.093	

T-TEST PAIRS=VAR00003 WITH VAR00005 (PAIRED)
  /CRITERIA=CI(.9500)
  /MISSING=ANALYSIS.


T-Test


Notes	
Output Created	01-DEC-2015 15:03:53	
Comments		
Input	Active Dataset	DataSet0	
	Filter	<none>	
	Weight	<none>	
	Split File	<none>	
	N of Rows in Working Data File	20	
Missing Value Handling	Definition of Missing	User defined missing values are treated as missing.	
	Cases Used	Statistics for each analysis are based on the cases with no missing or out-of-range data for any variable in the analysis.	
Syntax	T-TEST PAIRS=VAR00003 WITH VAR00005 (PAIRED)
  /CRITERIA=CI(.9500)
  /MISSING=ANALYSIS.	
Resources	Processor Time	00:00:00.00	
	Elapsed Time	00:00:00.01	


[DataSet0] 


Paired Samples Statistics	
	Mean	N	Std. Deviation	Std. Error Mean	
Pair 1	VAR00003	174.5740	10	146.39888	46.29539	
	VAR00005	206.5240	10	301.04037	95.19732	


Paired Samples Correlations	
	N	Correlation	Sig.	
Pair 1	VAR00003 & VAR00005	10	.586	.075	


Paired Samples Test	
	Paired Differences	
	Mean	Std. Deviation	Std. Error Mean	95% Confidence Interval of the Difference	
				Lower	
Pair 1	VAR00003 - VAR00005	-31.95000	245.81467	77.73342	-207.79522	

Paired Samples Test	
	Paired Differences	t	df	Sig. (2-tailed)	
	95% Confidence Interval of the Difference				
	Upper				
Pair 1	VAR00003 - VAR00005	143.89522	-.411	9	.691	

GRAPH
  /HISTOGRAM=VAR00003
  /PANEL ROWVAR=VAR00002 ROWOP=CROSS.


Graph


Notes	
Output Created	01-DEC-2015 15:09:43	
Comments		
Input	Active Dataset	DataSet0	
	Filter	<none>	
	Weight	<none>	
	Split File	<none>	
	N of Rows in Working Data File	20	
Syntax	GRAPH
  /HISTOGRAM=VAR00003
  /PANEL ROWVAR=VAR00002 ROWOP=CROSS.	
Resources	Processor Time	00:00:00.16	
	Elapsed Time	00:00:00.15	


[DataSet0] 


T-TEST PAIRS=VAR00003 WITH VAR00005 (PAIRED)
  /CRITERIA=CI(.9500)
  /MISSING=ANALYSIS.


T-Test


Notes	
Output Created	01-DEC-2015 15:19:28	
Comments		
Input	Active Dataset	DataSet0	
	Filter	<none>	
	Weight	<none>	
	Split File	<none>	
	N of Rows in Working Data File	20	
Missing Value Handling	Definition of Missing	User defined missing values are treated as missing.	
	Cases Used	Statistics for each analysis are based on the cases with no missing or out-of-range data for any variable in the analysis.	
Syntax	T-TEST PAIRS=VAR00003 WITH VAR00005 (PAIRED)
  /CRITERIA=CI(.9500)
  /MISSING=ANALYSIS.	
Resources	Processor Time	00:00:00.00	
	Elapsed Time	00:00:00.00	


[DataSet0] 


Paired Samples Statistics	
	Mean	N	Std. Deviation	Std. Error Mean	
Pair 1	VAR00003	12.1973	10	5.35404	1.69310	
	VAR00005	13.5190	10	7.06016	2.23262	


Paired Samples Correlations	
	N	Correlation	Sig.	
Pair 1	VAR00003 & VAR00005	10	.634	.049	


Paired Samples Test	
	Paired Differences	
	Mean	Std. Deviation	Std. Error Mean	95% Confidence Interval of the Difference	
				Lower	
Pair 1	VAR00003 - VAR00005	-1.32164	5.52795	1.74809	-5.27610	

Paired Samples Test	
	Paired Differences	t	df	Sig. (2-tailed)	
	95% Confidence Interval of the Difference				
	Upper				
Pair 1	VAR00003 - VAR00005	2.63282	-.756	9	.469	

T-TEST GROUPS=VAR00002('ESA' 'ThSA')
  /MISSING=ANALYSIS
  /VARIABLES=VAR00003
  /CRITERIA=CI(.95).


T-Test


Notes	
Output Created	01-DEC-2015 15:21:54	
Comments		
Input	Active Dataset	DataSet0	
	Filter	<none>	
	Weight	<none>	
	Split File	<none>	
	N of Rows in Working Data File	20	
Missing Value Handling	Definition of Missing	User defined missing values are treated as missing.	
	Cases Used	Statistics for each analysis are based on the cases with no missing or out-of-range data for any variable in the analysis.	
Syntax	T-TEST GROUPS=VAR00002('ESA' 'ThSA')
  /MISSING=ANALYSIS
  /VARIABLES=VAR00003
  /CRITERIA=CI(.95).	
Resources	Processor Time	00:00:00.02	
	Elapsed Time	00:00:00.00	


[DataSet0] 


Group Statistics	
	VAR00002	N	Mean	Std. Deviation	Std. Error Mean	
VAR00003	ESA	10	12.1973	5.35404	1.69310	
	ThSA	10	13.5190	7.06016	2.23262	


Independent Samples Test	
	Levene's Test for Equality of Variances	t-test for Equality of Means	
	F	Sig.	t	df	
					
VAR00003	Equal variances assumed	.005	.944	-.472	18	
	Equal variances not assumed			-.472	16.779	

Independent Samples Test	
	t-test for Equality of Means	
	Sig. (2-tailed)	Mean Difference	Std. Error Difference	95% Confidence Interval of the Difference	
				Lower	
VAR00003	Equal variances assumed	.643	-1.32164	2.80199	-7.20841	
	Equal variances not assumed	.643	-1.32164	2.80199	-7.23927	

Independent Samples Test	
	t-test for Equality of Means	
	95% Confidence Interval of the Difference	
	Upper	
VAR00003	Equal variances assumed	4.56513	
	Equal variances not assumed	4.59599	

T-TEST GROUPS=VAR00002('ESA' 'ThSA')
  /MISSING=ANALYSIS
  /VARIABLES=VAR00003
  /CRITERIA=CI(.95).


T-Test


Notes	
Output Created	01-DEC-2015 15:24:11	
Comments		
Input	Active Dataset	DataSet0	
	Filter	<none>	
	Weight	<none>	
	Split File	<none>	
	N of Rows in Working Data File	20	
Missing Value Handling	Definition of Missing	User defined missing values are treated as missing.	
	Cases Used	Statistics for each analysis are based on the cases with no missing or out-of-range data for any variable in the analysis.	
Syntax	T-TEST GROUPS=VAR00002('ESA' 'ThSA')
  /MISSING=ANALYSIS
  /VARIABLES=VAR00003
  /CRITERIA=CI(.95).	
Resources	Processor Time	00:00:00.02	
	Elapsed Time	00:00:00.00	


[DataSet0] 


Group Statistics	
	VAR00002	N	Mean	Std. Deviation	Std. Error Mean	
VAR00003	ESA	10	174.5740	146.39888	46.29539	
	ThSA	10	206.5240	301.04037	95.19732	


Independent Samples Test	
	Levene's Test for Equality of Variances	t-test for Equality of Means	
	F	Sig.	t	df	
					
VAR00003	Equal variances assumed	.479	.498	-.302	18	
	Equal variances not assumed			-.302	13.031	

Independent Samples Test	
	t-test for Equality of Means	
	Sig. (2-tailed)	Mean Difference	Std. Error Difference	95% Confidence Interval of the Difference	
				Lower	
VAR00003	Equal variances assumed	.766	-31.95000	105.85742	-254.34819	
	Equal variances not assumed	.768	-31.95000	105.85742	-260.58494	

Independent Samples Test	
	t-test for Equality of Means	
	95% Confidence Interval of the Difference	
	Upper	
VAR00003	Equal variances assumed	190.44819	
	Equal variances not assumed	196.68494	

*Nonparametric Tests: Independent Samples.
NPTESTS
  /INDEPENDENT TEST (VAR00003) GROUP (VAR00002)
  /MISSING SCOPE=ANALYSIS USERMISSING=EXCLUDE
  /CRITERIA ALPHA=0.05  CILEVEL=95.


Nonparametric Tests


Notes	
Output Created	01-DEC-2015 15:26:28	
Comments		
Input	Active Dataset	DataSet0	
	Filter	<none>	
	Weight	<none>	
	Split File	<none>	
	N of Rows in Working Data File	20	
Syntax	NPTESTS
  /INDEPENDENT TEST (VAR00003) GROUP (VAR00002)
  /MISSING SCOPE=ANALYSIS USERMISSING=EXCLUDE
  /CRITERIA ALPHA=0.05  CILEVEL=95.	
Resources	Processor Time	00:00:00.20	
	Elapsed Time	00:00:00.29	


[DataSet0] 
